# Extent of liver inflammation in predicting response to interferon α & Ribavirin in chronic hepatitis C patients: a cohort study

**DOI:** 10.1186/1471-230X-12-71

**Published:** 2012-06-14

**Authors:** Shirin Mirza, Amna Rehana Siddiqui, Saeed Hamid, Muhammad Umar, Shaheena Bashir

**Affiliations:** 1 Department of Community Health Sciences, Aga Khan University, Karachi, Pakistan; 2 Public Health Solutions Pakistan, Islamabad, Pakistan; 3 Department of Family and Community Medicine, College of Medicine, King Saud University, Riyadh, Saudi Arabia; 4 Department of Medicine, Aga Khan University, Karachi, Pakistan; 5 Department of Medicine, Holy Family Hospital, Rawalpindi, Pakistan; 6 Department of Mathematics and Statistics, McMaster University, Hamilton, Canada

## Abstract

**Background:**

Liver inflammation due to HCV infection leads to fibrosis, which is an independent predictor of treatment response to interferon therapy in Chronic Hepatitis C (CHC) patients. This relationship has not been studied for liver inflammation on pretreatment liver biopsy and End of Treatment Response (ETR). ALT is a less invasive test than liver biopsy for measuring liver inflammation. Aim of this study was to compare ETR to Interferon α (recombinant Interferon) & Ribavirin in CHC patients having higher and lower grades of liver inflammation and to determine the diagnostic accuracy of pretreatment ALT for grades of liver inflammation.

**Methods:**

A retrospective cohort of 876 naïve CHC patients, who completed Interferon α & Ribavirin for 24 weeks, was studied for ETR. Pretreatment grade of inflammation on liver biopsy was taken as the exposure variable. It was classified as high if there was moderate or severe and low if there was minimal or mild. Multivariable logistic regression modeling was performed. Diagnostic accuracy of pretreatment ALT for liver inflammation grades was determined by computing Area Under the Receiver Operator Curve (AUROC).

**Results:**

Of all patients, 672 having diagnostic liver biopsy and ETR available were analyzed. Among them, 103 had high and 569 had low grades of liver inflammation. Mean age was 36.9 (SD 9.1) years, with patients with high grades being older than those with low grades inflammation (p = 0.03). High grades of liver inflammation was associated with ETR (RR 1.17, 95% CI 1.12–1.18) adjusting for age, Total Leukocyte count (TLC) and pretreatment levels of ALT, irrespective of liver fibrosis. This relation remained significant for ‘bridging fibrosis and cirrhosis’ and not for ‘no’ or ‘portal fibrosis’. AUROC of pretreatment ALT for males and females was moderately accurate for severe inflammation compared to minimal inflammation and less accurate for high grades compared to low grades.

**Conclusions:**

ETR in patients with higher grades of liver inflammation was 17% higher than those with lower grades irrespective of fibrosis and 9% higher for bridging fibrosis and cirrhosis. Pretreatment ALT was moderately accurate for severe inflammation only on liver biopsy in both males and females.

## Background

Hepatitis C virus (HCV) is affecting 170 million people worldwide [1], 8 million in Pakistan alone [2]. The viral entry into the host activates T-helper cells as well as the cytotoxic T-cells for elaborating inflammatory cytokines in liver [3]. If the cellular immune response remains ineffective in clearance of the virus, it leads to hepatocellular injury depicted by chronic inflammation, and ultimately to liver fibrosis and cirrhosis [3]. Various quantitative grading systems are used to measure liver cells insult on biopsy; of these, modified Histological Index (HAI) [4] is commonly used in Pakistan. Serum Alanine aminotransferase (ALT) is a less invasive biochemical marker for indicating liver cell injury than liver biopsy.

In Pakistan, HCV genotype 3 is predominant with prevalence of 75–90%, from the six known HCV genotypes [5]. Standard therapy for naïve Chronis Hepatitis C (CHC) patients in this country is by Interferon α (recombinant/conventional Interferon) 3 million units subcutaneously thrice weekly and Ribavirin 800 mg/day for 24 weeks [6]. End of Treatment Response (ETR) to this therapy is 71–86.5% while Sustained Viral Response (SVR) is 52.3–86.3% [6].

This treatment costs about $ 800–1,000 per patient per month [6]. Given that health care is paid by individual patients, many CHC patients rely on government based subsidized treatment supposedly available in public hospitals; nevertheless majority have to pay by pocket for one or the other reason at many public and all of the private hospitals [6]. Pegylated Interferon is not administered to naïve CHC patients in Pakistan due to its high cost. Only the patients who do not respond (ETR negative: PCR for HCV RNA positive at end of 24 weeks of completion of treatment) to Interferon α (recombinant) and Ribavirin therapy are considered for Pegylated Interferon α & Ribavirin based on favorable predictors [6].

HCV genotypes 2 and 3, low viral load (< 2 million IU/ml), absence of cirrhosis, mild or absent portal fibrosis on pretreatment liver biopsy are favorable predictors for response to standard Interferon α and Ribavirin therapy [7-9]. American Association for the Study of Liver Disease (AASLD) practice guidelines recommend quantitative PCR for HCV RNA for quantification of viral load and HCV genotyping prior to antiviral therapy in CHC patients [10]; same has been proposed by the European Association for the Study of the Liver (EASL) [11]. These tests are not routinely done in Pakistan due to its high costs and lack of standardized procedures [6]. However, liver biopsy is performed to a larger extent at the HCV treatment clinics. According to the EASL guidelines published in 2011, liver biopsy is still the reference method for measuring liver inflammation and fibrosis, which tells about the liver disease severity prior to the therapy [11].

Few studies report whether histological variables other than fibrosis on liver biopsy, like grade of inflammation, can predict the response to antiviral therapy in CHC patients, showing conflicting results. Study by Daboul I et al. in Ohio, USA showed that response at 12 weeks (EVR positive) in CHC patients with high grades of liver inflammation was 60% compared to 68% in those with low grades when treated with Pegylated Interferon and Ribavirin [12]. Another study in the Middle East reported positive correlation between response rate after 24 weeks of stopping the therapy (SVR positive) and liver inflammation [13]. The former was conducted in HCV genotype 1 [12] and the latter in HCV genotype 4 patients [13]. Moreover, both studies have small sample size affecting the internal validity. Therefore relationship of ETR to Interferon α (recombinant/conventional Interferon) and Ribavirin therapy for 24 weeks with high and low grades of liver inflammation in CHC patients remains unknown for HCV genotype 3 patients.

Upper limit of normal (ULN) ALT levels for patients with liver inflammation was 40 U/L in males and 30 U/L in females [14]. The new upper limit of normal of ALT for identification of patients with minimal to mild liver inflammation are defined as 30 U/L in males and 19 U/L in females [14]. These results are based on investigations in Western population; and sensitivity and specificity at these new levels for minimal to mild liver inflammation are 76% and 97% respectively [14].

The primary objective of this study was to compare ETR in CHC patients with higher grades to those with lower grades of pretreatment liver inflammation. Our secondary objective was to determine diagnostic accuracy of ALT for pretreatment grades of inflammation ascertained by modified HAI for grading liver inflammation on liver biopsy in CHC patients.

## Methods

### Study design & setting

A retrospective cohort of 876 naïve CHC patients, who received Interferon α & Ribavirin for 24 weeks between March 1998 and June 2009 was assembled. Pre-treatment grade of liver inflammation was the exposure variable and ETR ascertained by qualitative PCR for HCV RNA at completion of 24 weeks of Interferon α (recombinant/conventional Interferon) 3 million units subcutaneously thrice weekly and Ribavirin 800 mg/day therapy was taken as the outcome variable. Data were collected from medical records of the CHC patients treated at Holy Family hospital (HFH) in Rawalpindi and Aga Khan University hospital (AKUH) in Karachi, using a pretested structured Questionnaire. HFH is a government tertiary care hospital affiliated to Rawalpindi Medical College, Rawalpindi, which besides catering patients from Rawalpindi and Islamabad, also provides care to patients coming from surrounding parts of major provinces of Pakistan namely Punjab, Khyber Pakhtunkhwa and Northern areas of Pakistan. Prime Minister’s programme for control of hepatitis B and C was initiated in this hospital in the second half of 2006, after which the patients of CHC started getting regular treatment free of cost (cost for 24 weeks Interferon α and Ribavirin therapy is paid by the government) as per guidelines of Pakistan society of gastroenterology at this liver clinic. AKUH is one of the advanced private tertiary care hospital in Karachi, which caters patients from the largest city of Pakistan, Karachi and the other two provinces namely Sind and Balochistan. CHC patients treated at AKUH have to pay for their 24 weeks Interferon α and Ribavirin therapy themselves (out of pocket), though some are sponsored by private, semi private and government employees for their treatment.

### Study population

Naïve CHC patients attending the above mentioned out patient department of medicine at the two hospitals, receiving Interferon α (recombinant/conventional Interferon) 3 million units subcutaneously thrice weekly and Ribavirin 800 mg/day therapy for 24 weeks after having pretreatment liver biopsy were included. Those with decompensated liver disease, liver failure, other liver pathologies (like hepatitis B, haemochromatosis, liver cancer etc.) and renal insufficiency were excluded.

### Study variables and data management procedures

Data were collected for demographic (age, gender), and biological characteristics; baseline hematological investigations like hemoglobin (Hb), total leukocyte count (TLC) and biochemical investigations (serum ALT, AST, bilirubin, alkaline phosphatase), HCV genotype, HCV viral load by quantitative HCV for RNA at the start of the therapy, liver inflammation and fibrosis at liver biopsy by modified HAI or Batts BLS, Early Viral Response (EVR) measured by qualitative PCR for HCV RNA at 12 weeks of therapy and ETR by a trained data collector on the Performa. Pretreatment grade of liver inflammation was graded on liver biopsy by using modified HAI [4]. Liver inflammation was defined as high grades if the patient had moderate (inflammation score 9–12) or severe inflammation (inflammation score 13–18). It was defined as low grades if the patient had minimal (inflammation score 1–4) or mild inflammation (inflammation score 5–8). ETR was measured by qualitative PCR for HCV RNA at 24 weeks of completion of Interferon α & Ribavirin therapy [6]. It was termed as ‘positive’ if qualitative PCR for HCV RNA was negative and ‘negative’ if qualitative PCR for HCV RNA was positive [6].

Manual of operation and key document were made for the training of the data collector. Rechecking of other data sources like pathology lab reports were done in case of missing data on the medical record file. Completeness and correct data entry was verified and logical consistency of the recorded data was checked by the Principal Investigator (PI) daily for all the forms. Data entry programme was developed in ‘Epi Info’ version 6.04 (CDC, USA) [15]. Two independent data entry operators entered the data. Errors in the two entries were checked by generating an ‘error list’ which showed an error of 0.26%. PI validated the values on ‘error list’ against those in the questionnaire. These corrections were then made in the entered data by the data entry operators. Further, the entire corrected database was validated by the PI against the questionnaires.

### Ethical considerations

Confidentiality of all the participants was maintained by assigning code to each of them. Identification information was accessible to the PI only. Study was conducted after ethical approval by Ethical Review Committee of the AKUH after receiving letter of collaboration from HFH (June 22, 2009 under ERC number 1260-CHS/ERC-09).

### Statistical methods

Sample size was calculated using the software ‘Sample size determination in health studies-A practical manual’ version 2.0 (WHO) [16]. Taking proportion of disease (non responders, Qualitative PCR for HCH RNA positive) in exposed (high grades of inflammation) at the end of therapy as 40% [12], anticipated Relative Risk (RR) as 1.5, level of significance as 5% and power as 80%, at least 196 patients were required in each inflammation group. Taking the ratio of High to Low grades of liver inflammation as 1:4 [13], and anticipated missing data as 10%, at least 137 patients were required with high grades and 545 required with low grades of liver inflammation.

Statistical analysis was done using SPSS version 14 [17]. Mean and Standard Deviation (SD) are reported for continuous variables having normal distribution; median and Inter Quartile Range (IQR) for continuous variables having skewed distribution. Proportions are reported for categorical variables. Student’s *t*-test (or Mann Whitney *U* test in case of skewed distribution) were used as tests of significance for comparing continuous variables between the patients having high grades of liver inflammation to those having low grades. Chi-square test (or Fisher exact test in case of cell count less than 5) was conducted to compare the difference of categorical variables between the two groups of liver inflammation. Patients who were eligible for the study but did not complete the Interferon α & Ribavirin therapy for 24 weeks were compared to those who completed it to account for differences by baseline characteristics to address selection bias. P-value of < 0.05 was considered significant. Univariable logistic regression model was built and variables with p-value on likelihood ratio test less than 0.25 or biologically significant, were considered for the multivariable analysis. As liver inflammation is one of the factors for liver fibrosis [3] and it is known that high fibrosis is a poor predictor of response to Interferon therapy [7-9], liver fibrosis may have had a confounding effect and therefore analysis was also performed stratified on liver fibrosis (no fibrosis, portal fibrosis, bridging fibrosis and cirrhosis). For discrete variable (liver fibrosis in this case), stratified analysis is equivalent to adjustment of covariate using regression analysis [18]. As the sample size was large in this study and sparse data problem was not encountered, thus stratified analysis was attempted. EVR was found to be significant but having unavailable information for more than 15% of patients, was not included in modeling further. Multivariable analysis was based on patients having information on all the study variables required for model building (significant on univariable analysis or having biological significance). Imputation for missing values was not attempted as it was computationally difficult. Moreover imputing the missing values of continuous variables like ALT, AST and TLC with averages preserves the sample mean but the co-variance structure is distorted to the extent that estimates are biased towards zero. If regression is used for imputation, observed correlations are inflated and biased away from zero [19]. Unadjusted and adjusted Relative Risk (RR) and their 95% Confidence Interval (CI) were calculated. Interactions were explored between variables and confounding effects were checked, Hosmer and Lemeshow goodness of fit statistic was applied after checking for the co-variate pattern. P-value of >0.05 indicates that the model fits adequately.

Mean (SD) of pretreatment ALT (normally distributed) was computed for minimal, mild, moderate and severe liver inflammation group separately for males and females. Sensitivity and specificity of pretreatment ALT for mild, moderate and severe inflammation were calculated compared to minimal inflammation at deciles of ALT. Receiver operating characteristic (ROC) curves were plotted and Area Under the Receiver Operator Curve (AUROC) computed. ROC curves were also plotted for high grades of liver inflammation taking low grades liver inflammation as the reference category separately for males and females. Based on AUROC, accuracy of pretreatment ALT for the grade of inflammation was defined as non-informative (AUC = 0.5), less accurate (0.5 < AUC ≤ 0.7), moderately accurate (0.7 < AUC ≤ 0.9), highly accurate (0.9 < AUC < 1) and perfect (AUC = 1) [20]. Cut off values of pretreatment ALT for mild, moderate and severe inflammation compared to minimal inflammation, and high grades liver inflammation compared to low grades was decided based on optimal level of sensitivity and specificity i.e. the upper left most part of the ROC curve [20].

## Results

**Figure 1:**
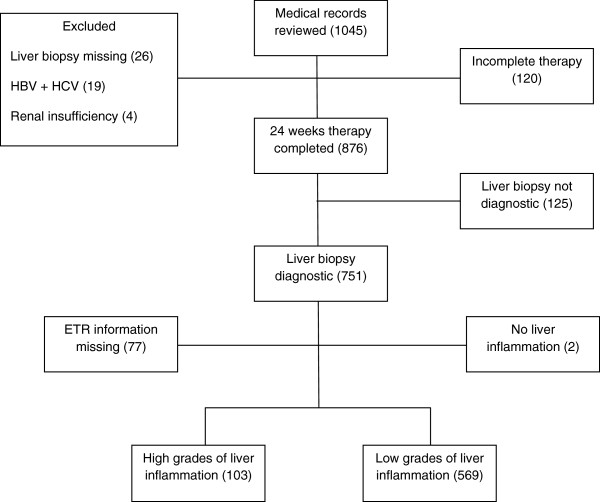
**Flow Diagram of the Chronic Hepatitis C patients at each stage of the study.** This flow diagram indicates number of patients (mentioned in brackets) at each stage of this retrospective cohort study.

A total of 1,045 records were screened, of which 672 patients completed the 24 week Interferon α and Ribavirin therapy and had diagnostic liver biopsy (Figure 1). One hundred and twenty patients did not complete the 24 week Interferon α & Ribavirin therapy. These patients had significantly higher Hb, TLC and bilirubin compared to those who completed the therapy (Table 1). There was no difference in grades of liver inflammation (p = 0.26) nor liver fibrosis (p = 0.57) between the patients who did not complete the therapy and those who completed it. Genotype data for HCV was available in 101 patients; 94% of them had genotype 3. Distribution of HCV genotypes did not vary between the patients who did not complete the therapy and those who completed it (p = 0.85). Quantitative PCR for HCV RNA prior to therapy was available in 141 patients, median 513,866 IU/mL (IQR 493–79,121,294 IU/mL). The patients who completed the 24 week Interferon α & Ribavirin therapy did not differ significantly from those who did not complete it with regards to the viral load (median 508,551 IU/mL vs. 594,217 IU/mL, p = 0.30) (Table 1).

**Table 1:** Baseline characteristics of patients who did not complete the therapy and those who completed it

**Study variable**	**Did not complete treatment**		**Completed treatment**		**P-value**
	**n**_**L**_	**Response to variable**	**n**_**C**_	**Response to variable**	
**Age**: n (%)	120		876		
< 40 year		79 (65.8)		534 (61.0)	0.31^a^
≥ 40 year		41 (34.2)		342 (39.0)	
**Gender**: n (%)	120		876		0.20^a^
Male		65 (54.2)		417 (47.6)	
Female		55 (45.8)		459 (52.4)	
**Marital Status**: n (%)	120		876		0.46^a^
Single		18(15.0)		107(12.2)	
Married		102(85.0)		769(87.8)	
**HCV genotype** n (%)	10		91		0.85 ^b^
Genotype 1		0 (0)		1 (1.1)	
Genotype 2		1 (10)		3 (3.3)	
Genotype 3		9 (90.0)		86 (94.5)	
Genotype 4		0 (0)		1 (1.1)	
**Viral load in IU/mL**(median(IQR))	16	594,217(16,481–29,445,155)	125	508,551(493–79,121,294)	0.30 ^c^
**ALT in U/L**(median(IQR))	108	79.5(16–957)	796	72(11–781)	0.45^c^
**AST in U/L** (median(IQR))	21	69(23–230)	151	46(14–508)	0.03^c^
**Bilirubin in mg/dL**(mean ± SD)	82	0.99 ± 0.44	553	0.89 ± 0.40	0.05^d^
**Alkaline Phosphatase** in U/L (mean ± SD)	82	218.8 ± 77.8	535	207.4 ± 98.7	0.31^d^
**Albumin in g/dL** (mean ± SD)	23	4.3 ± 0.4	170	4.2 ± 0.6	0.32^d^
**Alpha Fetoprotein in ng/mL** (median(IQR))	6	2.18(0.5–10.4)	63	2.4(0.2–16.5)	0.85^c^
**Hb in g/dL** (mean ± SD)	116	15.4 ± 14.2	826	13.7 ± 5.07	0.01^d^
**TLC in cell × 10**^**9**^**/L**(mean ± SD)	114	8.7 ± 8.8	805	7.5 ± 2.6	0.002^d^

^a^-Chi-square test, ^b^-Fisher’s exact test, ^c^-Mann Whitney-*U* test, ^d^-Student’s *t*-test; *n*_*L*_: no. of patients who did not complete treatment, *n*_*C*_: no. of patients who completed treatment.

Patients with high grades of liver inflammation were older than those with low grade (Table 2). The two groups did not differ with regards to gender distribution (Table 2). Mean Hb and mean pretreatment ALT were significantly higher while mean albumin was significantly lower in those with high grade liver inflammation compared to low grade (Tables 2 and 3). High grade inflammation group significantly differed from those with low grade with respect to liver fibrosis: no fibrosis (18.4% vs. 58.2%), portal fibrosis (35.9% vs. 33%), bridging fibrosis (20.4% vs. 7.6%) and cirrhosis (25.2% vs. 1.2%) respectively, depicting a p-value of <0.01.

**Table 2:** Demographic characteristics and pre-treatment blood CP of patients with high and low grades of inflammation

**Study variable**	**High grades of inflammation**		**Low grades of inflammation**		**P- Value**
	**n**_**1**_	**Response to variable**	**n**_**o**_	**Response to variable**	
**Demographic Characteristics**					
**Age**					0.031^a^
< 40 years	53	51.4%	357	62.7%	
≥ 40 years	50	48.5%	212	37.2%	
**Gender**					0.94^a^
Males	48	46.6%	263	46.2%	
Females	55	53.4%	306	53.8%	
**Pre-treatment Blood CP**					
**Hb in g/dL** (mean ± SD)	94	15.0 ± 4 .1	540	13.6 ± 1.9	0.025^b^
**TLC in cell × 10**^**9**^**/L** (mean ± SD)	92	7.5 ± 2.4	527	7.5 ± 2.0	0.882^b^

^a^-Chi-square test,^b^-Student’s *t*-test; *n*_*1*_: number of patients in High inflammation group, *n*_*o*_: number of patients in low inflammation group.

**Table 3:** Pre-treatment biochemical tests of patients with high and low grades of liver inflammation

**Study variable**	**High grades of inflammation**		**Low grades of inflammation**		**P- Value**
	**n**_**1**_	**Response to variable**	**n**_**o**_	**Response to variable**	
**Pre-treatment Biochemical tests**					
**ALT in U/L** (mean ± SD)	87	119.2 ± 76.6	508	85.6 ± 66.6	<0.01 ^a^
**AST in U/L** (median & IQR)	17	69 (22–229)	104	42 (14–508)	0.17^b^
**Bilirubin in mg/dL** (mean ± SD)	62	0.9 ± 0.3	370	0.9 ± 0.4	0.67^a^
**Alkaline phosphatase in U/L** (mean ± SD)	61	203 ± 131.6	356	207.3 ± 98.7	0.78^a^
**Albumin in g/dL** (mean ± SD)	29	3.0 ± 0.4	110	4.1 ± 0.4	0.04^a^

^a^- Student’s *t*-test,^b^- Mann Whitney *U* test, *n*_*1*_: number of patients in High inflammation group, *n*_*o*_: number of patients in low inflammation group.

Univariable logistic regression showed that, age < 40 years, male gender and EVR positive was positively associated with ETR, while increase in TLC was negatively associated with ETR positive (Table 4). Multivariable analysis depicted that ETR positive was 1.17 times (95% CI 1.12–1.18) higher in high grades group compared to those with low grades of liver inflammation; adjusting for age, pretreatment TLC and pretreatment ALT (Table 5). Hosmer and Lemeshow test for goodness of fit showed chi – square statistics as 6.23 (p-value 0.621) implying that the model fits well. Stratified analysis showed that ETR positive was 1.09 times (95% CI 1.02–1.19) higher in high grades group compared to those with low grades for patients having bridging fibrosis and cirrhosis. The association of ETR with grade of liver inflammation was neither significant in those having no fibrosis (RR 0.97, 95% CI 0.68–1.07) nor in those having portal fibrosis (RR 1.02, 95% CI 0.95–1.03). These latter three models were adjusted for age, pretreatment TLC and pretreatment ALT.

**Table 4:** Relationship between grades of inflammation and End of Treatment Response (ETR) by Unadjusted RR

**Variable**	**n**	**RR**	**95% CI RR**
**Grade of inflammation**:			
Low grades (Reference)	569	1	–
High grades	103	1.05	0.97–1.09
**Age**:			
<40 years	410	1.11	1.08–1.15
≥40 years (Reference)	262	1	–
**Gender**:			
Female (Reference)	361	1	–
Male	311	1.03	1.02-1.04
**Pretreatment TLC cells × 10**^**9**^**/L** (1 unit rise)	619	0.98	0.98–0.99
**Pretreatment ALT in U/L** (5 unit rise)	595	1	0.99–1.001
**Early viral response(EVR)**			
Negative (Reference)	92	1	–
Positive	250	1.6	1.4–1.8

*RR*: Risk Ratio.

**Table 5:** Relationship between grades of inflammation and End of Treatment Response by adjusted RR (N = 552)

**Variable**	**n**	**RR**	**95% CI RR**
**Grade of inflammation**			
Low grades (Reference)	472	1	–
High grades	80	1.17	1.12–1.18
**Age**			
< 40 years	334	1.16	1.11–1.23
≥ 40 years (Reference)	218	1	–
**TLC**			
< 7.2 cells × 10^9^/L	266	1.14	1.10–1.17
≥7.2 cells × 10^9^/L (Reference)	286	1	–
**ALT in U/L** (5 unit rise)	552	1	0.99–1.01

*RR*: Risk Ratio.

**Figure 2:**
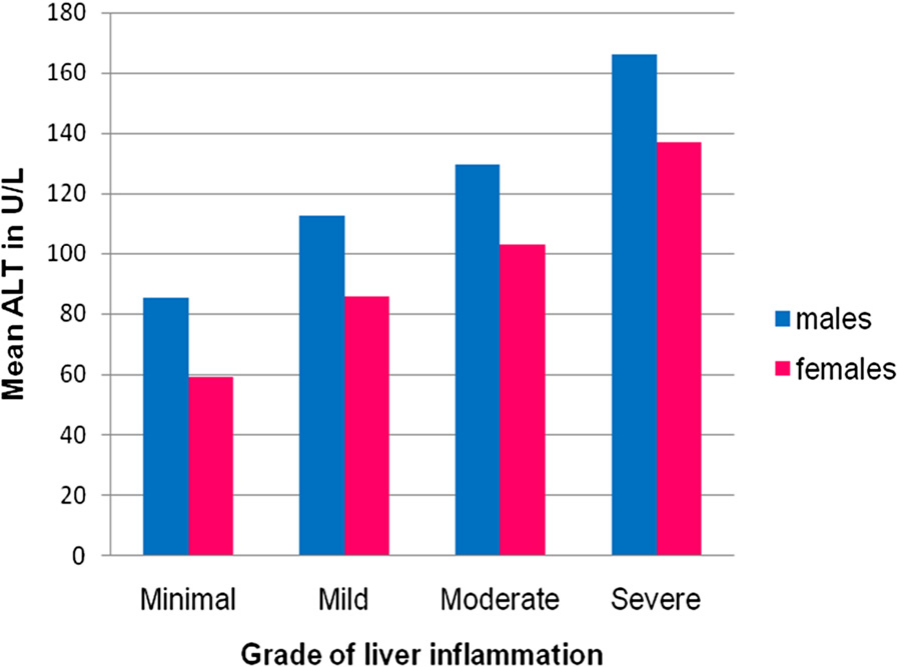
**Mean level of pre-treatment ALT in male and females for various grade of liver inflammation.** This figure depicts that as liver inflammation increases, ALT level rises. ALT levels are higher for males as compared to the females at all grades of liver inflammation.

**Figure 3:**
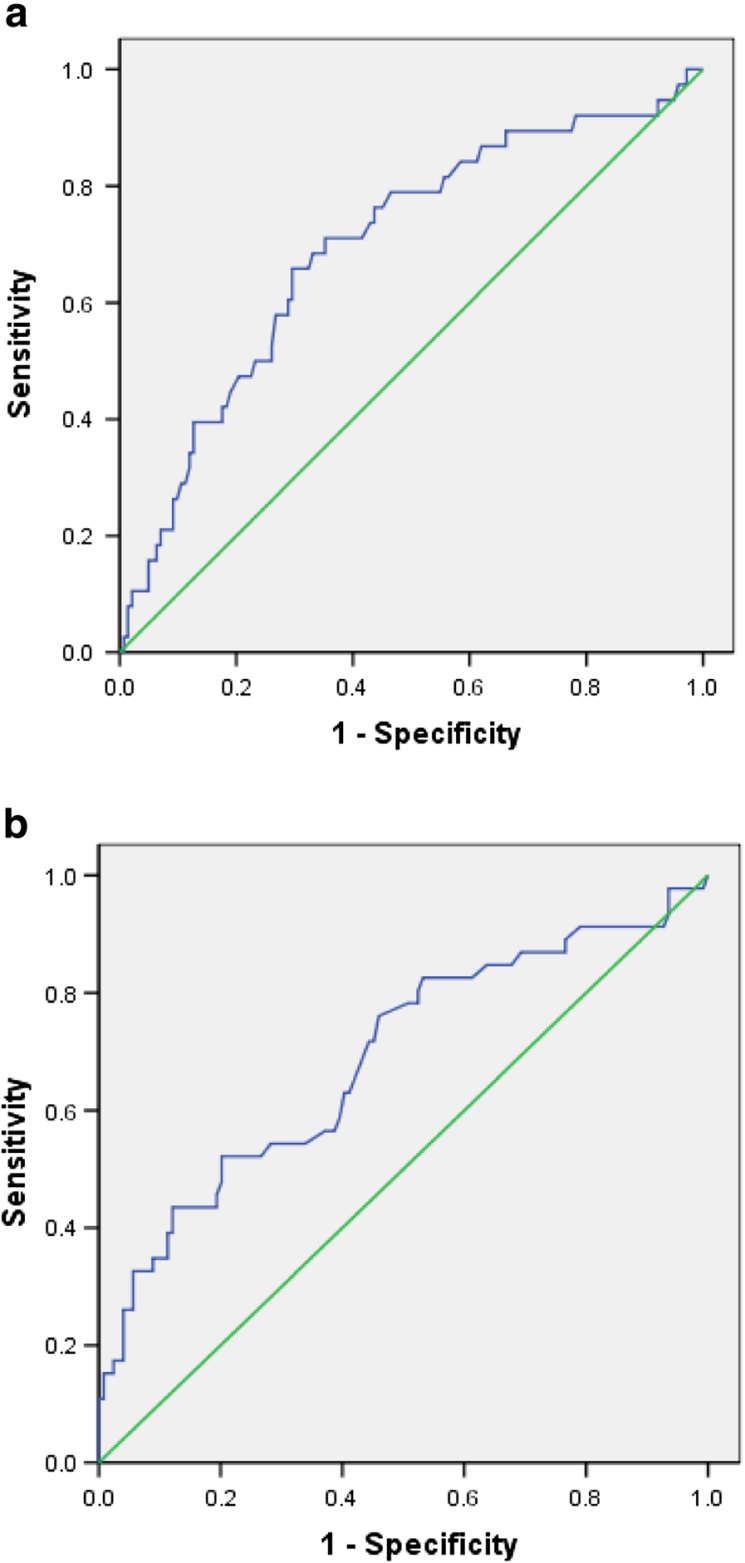
**ROC curve for validity of pretreatment ALT for severe vs. minimal inflammation.** Part ‘**a**’ shows AUROC of pre-treatment ALT for severe compared to minimal liver inflammation in males, having a value of 0.871. Part ‘**b**’ shows AUROC of pre-treatment ALT for severe compared to minimal liver inflammation in females, a value of 0.744. Pre-treatment ALT is moderately accurate in measuring severe as compared to minimal liver inflammation in both genders, more in males as compared to the females.

**Figure 4:**
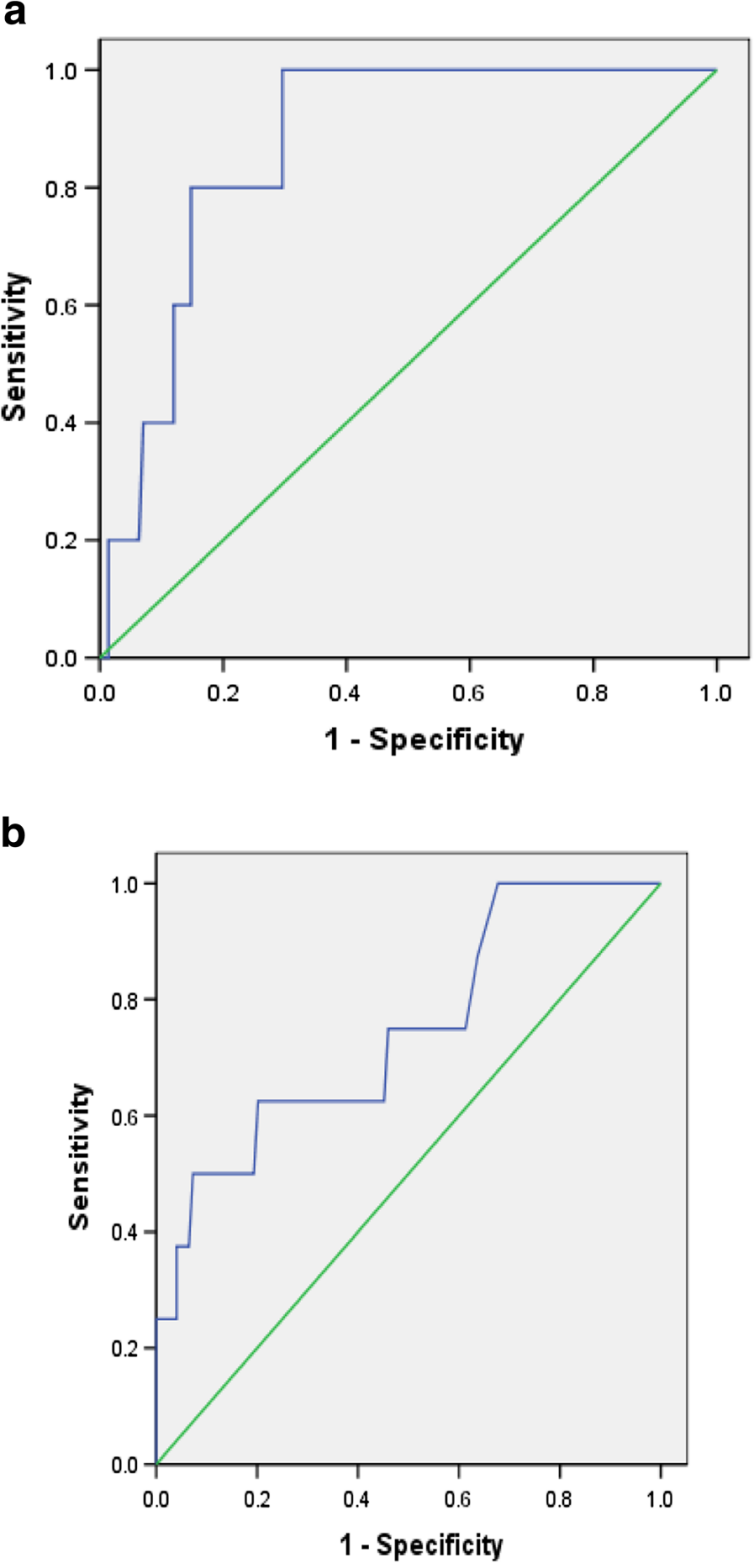
**ROC curve for validity of pretreatment ALT for mild vs. minimal inflammation.** Part ‘**a**’ shows AUROC of pre-treatment ALT for mild compared to minimal liver inflammation in males, having a value of 0.629. Part ‘**b**’ shows AUROC of pre-treatment ALT for mild compared to minimal liver inflammation in females, a value of 0.638. Pre-treatment ALT is less accurate in measuring mild as compared to minimal inflammation in both genders.

**Figure 5:**
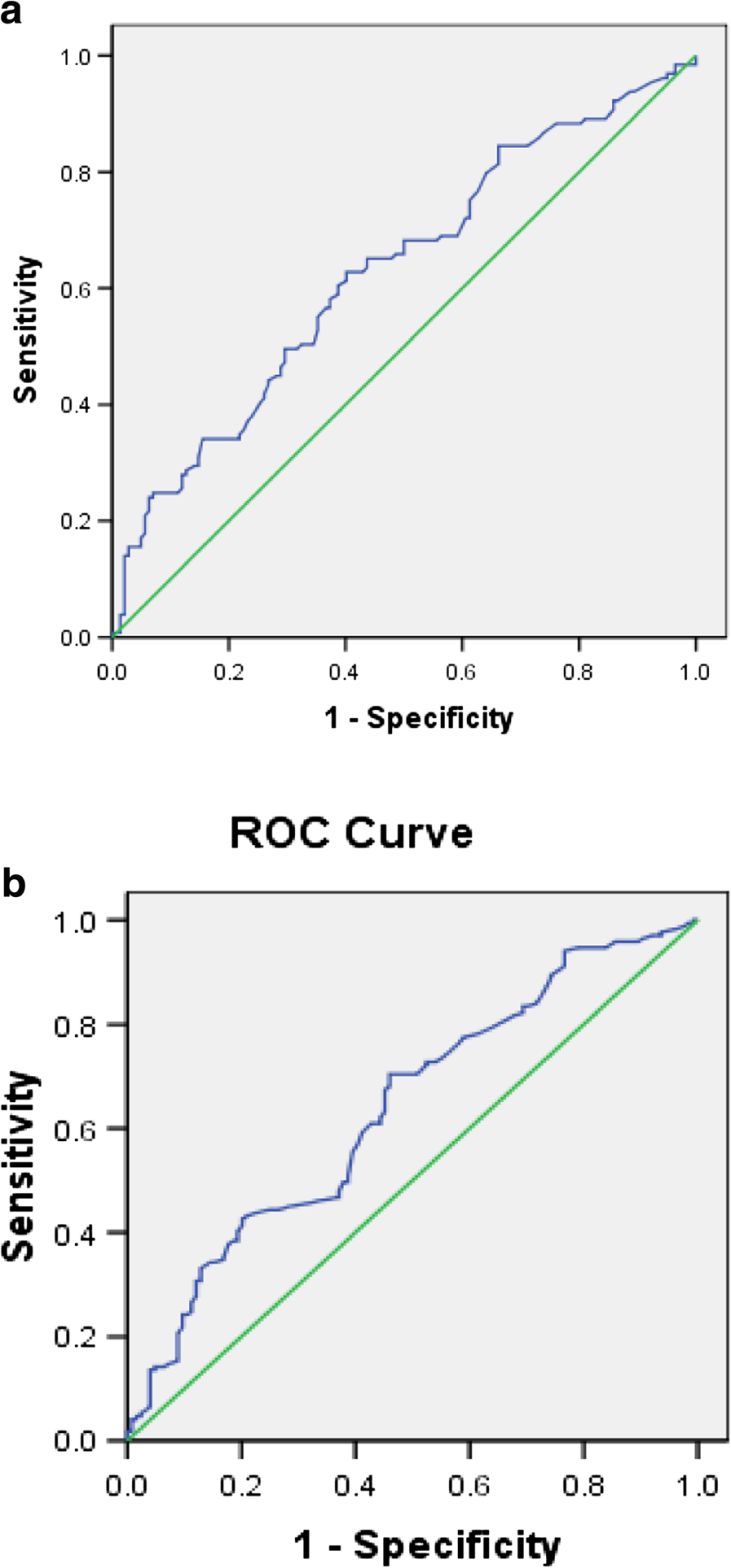
**ROC curve for validity of pretreatment ALT for moderate vs. minimal inflammation.** Part ‘**a**’ shows AUROC of pre-treatment ALT for moderate compared to minimal liver inflammation in males, having a value of 0.696. Part ‘**b**’ shows AUROC of pre-treatment ALT for moderate compared to minimal liver inflammation in females, a value of 0.688. Pre-treatment ALT is less accurate in measuring moderate as compared to minimal inflammation in both genders.

Mean levels of pretreatment ALT increased as the liver inflammation increased and was less in females as compared to males for all inflammation groups (Figure 2). AUROC showed that pretreatment ALT was moderately accurate for severe inflammation in both males and females (Figure 3). It did not correlate well with mild and moderate liver inflammation (Figures 4 and 5). Taking low grades of liver inflammation as the reference category, pretreatment ALT was less accurate (AUROC = 0.67) in males as well as in females (AUROC = 0.64) for high grades of liver inflammation.

According to the optimization of sensitivity and specificity, cut-off levels of 53 U/L, 65 U/L and 75 U/L indicated mild, moderate and severe liver inflammation respectively in females. While cut-off levels of 68.4 U/L, 85 U/L and 118 U/L can be used to indicate mild, moderate and severe liver inflammation in males respectively. In females pretreatment ALT level of 71 U/L indicates high grades liver inflammation while a value of 86.5 U/L indicates high grades of liver inflammation in males.

## Discussion

Higher grades of inflammation (moderate or severe) on pretreatment liver biopsy, younger age (<40 years) and TLC count of less than 7.2 cells × 10^9^/L before initiation of therapy were independent predictors of positive ETR in naïve CHC patients. Pretreatment ALT is a moderately accurate test for indicating severe inflammation on pretreatment liver biopsy in both male and female CHC patients; who have not been previously treated for chronic HCV infection.

The relationship between higher grades of inflammation and positive ETR is consistent with the finding of Derbala MF et al. who reported positive correlation (p-value < 0.05) between higher grades of inflammation and response rate (SVR positive) in HCV genotype 4 patients treated with Pegylated Interferon and Ribavirin for one year [13]. These finding are also biologically plausible. HCV activates the cytotoxic T-lymphocytes which releases inflammatory cytokines that causes liver inflammation in CHC patients [21]. Grade of liver inflammation correlates with the underlying immune response of the host i.e. higher the immune response to HCV infection, higher is the liver inflammation [22]. Interferon α is a cytokine that has two modes of action: direct antiviral affect as well as it acts as an immunomodulator to clear HCV infection [23]. Therefore high grades of liver inflammation depicts higher immune response to HCV which responds more to immune modulation effect of Interferon α compared to low grades of inflammation. But this finding remains true for patient with bridging fibrosis and cirrhosis only as found in our study.

The two inflammation groups varied significantly with regards to age; and this finding is also similar to a study conducted in China which showed that increasing age correlated with the inflammatory activity (p-value < 0.05) [24]. Literature supports the positive relationship of younger age (< 40 years) and positive ETR. Poynard T et al. in their study showed that SVR positive to Interferon α-2b and Ribavirin for duration of 48 weeks in patients ≤ 40 years was 48% compared to 34% in those more than 40 years of age [25]. Young age at the start of treatment was significantly positively associated with response to combination therapy of Interferon α and Ribavirin in other studies as well [23]. We also noted that baseline characteristics of the CHC patients who completed Interferon α and Ribavirin therapy for 24 weeks in this study are comparable to the CHC patients in Pakistan. Mean age of patients was 36.8 (SD 9.1) years which is comparable to a study by Zuberi BF in Karachi which reported mean age of males as 35.9(SD8.0) years and that in females as 39.1 (SD 9.1) years [26].

Serum albumin level in patients with high grades liver inflammation was significantly less than those in low grades of inflammation group . This can be explained by strong positive correlation of liver inflammation with stage of fibrosis (correlation coefficient gamma = 0.724). When inflammation grade increases, fibrosis increases and serum albumin level decrease [27].

According to a study by Davis GL et al., TLC was a potential predictor of response to recombinant Interferon therapy at univariable analysis (p-value < 0.15), along with dose of recombinant Interferon, weight, and body surface area, ongoing use of ethanol and presence of symptoms [28]; at multivariable analysis only dose of recombinant Interferon was a predictor of response [28]. Findings of the current study is contrary to that of the above as TLC of less than 7.2 × 10^9^ cells/L had independent positive association with positive ETR adjusting for age, grade of liver inflammation and pretreatment ALT at the multivariable analysis. Reasons for this variation need further investigation.

Increase in the grade of liver inflammation, tended to correlate with increasing mean levels of pretreatment ALT. This finding is similar to a study conducted in Iran in which the median score of modified HAI was lower in patients with normal ALT (defined as ≤ 49 U/L) compared to those with elevated ALT level (median score of modified HAI 5 vs. 6, p-value: 0.001) [29]. This indicates that serum ALT levels could be used to point towards the increasing grade of liver inflammation. However, to be used with caution as ROC curve analysis in this study showed that ALT is moderately acceptable test for severe liver inflammation.

One of the strengths of this study is its retrospective design which made it resource efficient. Data source of this study were medical records and laboratory reports which is an efficient way of gaining information and answering the research question. Much of the variables studied were laboratory, radiology, and pathology based; hence more objective compared to other data available in records; as lab based tests are done consistently for all and are readily available. The results obtained in this study could be a result of chance; though unlikely. This is supported by the precise 95% confidence intervals.

An important issue that may have affected the results is loss to follow-up bias as 12%(n = 120) of the CHC patients in eligible cohort did not complete the 24 week therapy and hence were excluded; had they responded positively (RR 1.14, 95% CI 1.14–1.20) or negatively (RR 1.18, 95% CI 1.14–1.20) to 24 weeks therapy, our results may not have changed. We are confident that loss to follow-up bias was not substantial although they differed on levels of Hb, TLC and bilirubin significantly.

Another source of measurement errors could be related to assessment of liver biopsy by different pathologists. Study by Westin J et al. showed 95–96% agreement between 3 pathologists grading liver biopsy on the basis of modified HAI [30]. In the current study, 267 biopsies were reported by a single pathologist; hence we evaluated this subgroup of patients’ data for relationship of positive ETR and grades of liver inflammation; which obviously was not significant (RR 1.09, 95% CI 0.93–1.14); due to the small insufficient sample size (post hoc power reduced to 43.5% for subgroup analysis); however the direction of relationship tended to be along with the study results. Nevertheless interobservor agreement for reporting inflammation grades of liver could not be ruled out in this study.

Clinically, SVR is more relevant in following response to Interferon α and Ribavirin therapy than ETR. The patients with ETR positive are more than 20 times likely to have SVR positive [31]. This study was aimed to see the relationship of liver inflammation with ETR in naïve CHC patients so data regarding SVR was not gathered. Given the positive relationship between the two (higher the liver inflammation, higher the ETR- in those having bridging fibrosis and cirrhosis), future study will be conducted to explore the relationship between liver inflammation and SVR.

Of all the patients in whom HCV genotype data was available (n = 101), 1.1% had HCV genotype 1 who require 48 weeks Interferon therapy instead of 24 weeks. Thus the response in these patients may have been less as compared to those having HCV genotype 2 or 3. But as HCV genotype is not associated with liver inflammation [32], it may not have confounded the relationship between the liver inflammation and ETR.

HCV genotype 3 prevalence as found in this study is consistent with the reported proportion of HCV genotype 3 as 87. 8% in a study conducted by Mumtaz K et al. in Karachi in 2008 [33]. Approximately 5% patients in the current study had cirrhosis on liver biopsy. Idrees M et al. in their multicentre study in Pakistan reported the proportion of patients with cirrhosis on liver biopsy as 4% [34]. Including the similarity in mean age of population with other studies as mentioned above shows that patient population of this study is reasonably representative of HCV patients in Pakistan; and therefore could be generalized for the target population.

## Conclusions

This study shows that treating physicians should start treatment of CHC patients at an early age for favorable response. Patients having higher grades of liver inflammation (moderate or severe) will respond more to the Interferon α and Ribavirin therapy. Though liver biopsy is not done routinely, higher levels of serum ALT can point towards severe inflammation. Other liver function tests like low levels of serum albumin also reflect high grades of liver inflammation.

## Competing interest

All authors declare that they have no competing interests.

## Authors’ contributions

SM designed the study and collected the data. SM, ARS, SH, MU and SB interpreted the data. SM and ARS wrote and revised the manuscript for logical flow and consistency. SH and MU provided clinical inputs while SB provided statistical inputs. ARS supervised the project. All authors approved the final manuscript.

## Pre-publication history

The pre-publication history for this paper can be accessed here:

http://www.biomedcentral.com/1471-230X/12/71/prepub
